# Neighbourhood Characteristics and Long-Term Air Pollution Levels Modify the Association between the Short-Term Nitrogen Dioxide Concentrations and All-Cause Mortality in Paris

**DOI:** 10.1371/journal.pone.0131463

**Published:** 2015-07-21

**Authors:** Séverine Deguen, Claire Petit, Angélique Delbarre, Wahida Kihal, Cindy Padilla, Tarik Benmarhnia, Annabelle Lapostolle, Pierre Chauvin, Denis Zmirou-Navier

**Affiliations:** 1 EHESP School of Public Health, Rennes, France; 2 INSERM U1085 (IRSET), Rennes, France; 3 INSERM U707, Research Group on the Social Determinants of Health and Healthcare, Paris, France; 4 Lorraine University Medical School, Vandœuvre-les-Nancy, France; Hasselt University, BELGIUM

## Abstract

**Background:**

While a great number of papers have been published on the short-term effects of air pollution on mortality, few have tried to assess whether this association varies according to the neighbourhood socioeconomic level and long-term ambient air concentrations measured at the place of residence. We explored the effect modification of 1) socioeconomic status, 2) long-term NO_2_ ambient air concentrations, and 3) both combined, on the association between short-term exposure to NO_2_ and all-cause mortality in Paris (France).

**Methods:**

A time-stratified case-crossover analysis was performed to evaluate the effect of short-term NO_2_ variations on mortality, based on 79,107 deaths having occurred among subjects aged over 35 years, from 2004 to 2009, in the city of Paris. Simple and double interactions were statistically tested in order to analyse effect modification by neighbourhood characteristics on the association between mortality and short-term NO_2_ exposure. The data was estimated at the census block scale (n=866).

**Results:**

The mean of the NO_2_ concentrations during the five days prior to deaths were associated with an increased risk of all-cause mortality: overall Excess Risk (ER) was 0.94% (95%CI=[0.08;1.80]. A higher risk was revealed for subjects living in the most deprived census blocks in comparison with higher socioeconomic level areas (ER=3.14% (95%CI=[1.41-4.90], p<0.001). Among these deprived census blocks, excess risk was even higher where long-term average NO_2_ concentrations were above 55.8 μg/m^3^ (the top tercile of distribution): ER=4.84% (95%CI=[1.56;8.24], p for interaction=0.02).

**Conclusion:**

Our results show that people living in census blocks characterized by low socioeconomic status are more vulnerable to air pollution episodes. There is also an indication that people living in these disadvantaged census blocks might experience even higher risk following short-term air pollution episodes, when they are also chronically exposed to higher NO_2_ levels.

## Introduction

The association between ambient air pollution and mortality is widely documented in the literature [1,2]. Various pollutants were studied, including Particulate Matter (PM) and nitrogen dioxide (NO_2_). Times-series and case-crossover studies were the most frequently used study designs for assessment of the short-term effects of exposure to air pollution, whereas cohort studies were most commonly performed to evaluate long-term effects. Long-term exposures have greater effects than short-term variation of pollutants’ concentrations [3–5]. In the APHENA multi-city study, the combined effect of PM_10_ on all-cause mortality across all-ages for cities with daily air pollution data ranged from 0.2% to 0.6% for a 10 μg/m^3^ increase in ambient PM_10_ concentrations [5]. However, the public health significance of these results is still a matter of debate because of the harvesting (or death displacement) effect [6–8]. People at high risk of dying at any given point in time may die prematurely due to an air pollution episode, but these precipitated deaths are followed by a short period of decline in mortality—during which the pool of people at high risk of dying is reconstituted. The reconstitution of this pool is influenced by many factors such as age, smoking, pre-existing diseases, poor socioeconomic conditions, and chronic exposure to air pollution. Little research has been undertaken to explore the joint impact of short-term variations in air pollution and of chronic exposure. Such research as there is makes an underlying assumption that the effect of short-term variations in exposure is constant over the range of long-term exposures [5]. However, acute outcomes associated with short-term variations in air quality build up upon chronic conditions that have been shown to be linked with long-term exposures—in particular such cardiovascular conditions as haemostasis impairment, atherosclerosis or hypertension [9, 10].

Besides, deprived populations are reported to be more affected by exposure to air pollution [11–13] via two main pathways: differential exposure (disadvantaged groups may be more intensely exposed to pollution than affluent groups) and differential vulnerability (disadvantaged people may be more likely to manifest the adverse effects of air pollution because, in general, they already experience poorer health status due to social determinants). However, though several studies have assessed whether socioeconomic status (SES) modifies the risk of all-cause mortality in relation to short-term variations in air pollution, the results remain inconsistent: some studies have observed higher risk among subjects with a low SES [14–18] whereas others did not [19–22]. The variety of indicators defining socioeconomic status could partially explain the difference in results observed: income [22], education level [15–17, 21], housing characteristics [19], occupational activities [16, 17, 19], and sometimes a global socioeconomic index encompassing an array of variables [18, 22, 23]. Moreover, these SES measures were assessed at different levels across studies, using either individual [16, 19, 21] or area-based measures [17, 18, 22, 23].

In this context, the main objective of the present study was to explore how a combination of neighbourhood characteristics (socioeconomic profile and long-term exposure to ambient air NO_2_ levels) might modify the short-term association between variations in NO_2_ and all-cause mortality in Paris (France). Paris is characterized by long-term average NO_2_ concentrations that vary substantially across the city according to traffic density (the main determinant of NO_2_ levels) as well as by a variety of districts hosting populations with contrasting socioeconomic profiles.

## Material and Methods

### Study setting and small-area level

The city of Paris has a population of about 2,250,000. The small-area level used was the IRIS (a French acronym for ‘blocks for incorporating statistical information’). Designed by the French National Census Bureau (INSEE), the IRIS constitutes the smallest census unit area whose aggregate data can be used on a routine basis. The city of Paris is subdivided into 992 IRIS—named “census blocks” hereafter—with a mean population of 2,199 inhabitants and a mean area of 0.11 km^2^ (range from 0.009 to 5.4 km^2^).

### Health data

We considered all deaths that occurred in the city of Paris for residents older than 35 years old from January 2004 to December 2009. All-cause mortality data were provided by the death registry of the city of Paris. Individual information on age, sex, date of death, and census block of residence was available for each case. For confidentiality reasons it was not possible to distinguish causes of mortality, thus external causes of deaths could not be excluded. The analysis included only subjects older than 35 years at the time of death to minimize this bias since accidental causes of death are dominant in subjects under 35 years old [24]. We obtained the population size in each stratum from the INSEE. Ethical approval was obtained from the French commission on data privacy and public liberties (CNIL—Commission Nationale de l’Informatique et des Libertés).

### Air pollution data

NO_2_ was selected as the exposure indicator because it is recognized to be a good tracer of the air pollution generated by traffic, the main source of air pollution in Paris, and to present a small scale spatial heterogeneity greater than other pollutants.

- Estimation of long-term NO_2_ concentrations at the census blocks level: Annual NO_2_ concentrations were modelled from a grid of 25x25m resolution throughout the period 2002–2009 by engineers at AirParif (the regional association for the surveillance of air quality: http://www.airparif.asso.fr/). The ESMERALDA inter-regional platform for air quality mapping and forecasting (www.esmeralda-web.fr) provided background pollution data, while the STREET dispersion model [25] was used for traffic-related pollution. To compute annual NO_2_ concentrations at a fine spatial resolution (25x25m), these models incorporated several input data types: emission inventories, meteorological data and background pollution measurements—supplied respectively by the industry, by regional environmental administration, by Météo-France (the French meteorological agency) and by the regional network monitoring stations, alongside building and traffic parameters. Air pollutant concentrations were then aggregated at census block scale in order to obtain annual mean NO_2_ concentration for each census block. Long-term NO_2_ concentrations at census block scale were computed as the mean of daily NO_2_ concentrations from 2002 to 2009. The small spatial heterogeneity of the long-term NO_2_ concentrations lead us to categorize the exposure into tertiles of the distribution (limits for each interval are shown in Table 1)
- Estimation of short-term NO_2_ concentrations and exposures: To reconstitute census-block-specific NO_2_ daily variability within each census block, we combined the annual NO_2_ concentrations estimated by the dispersion models described in the previous section with the daily NO_2_ concentrations measured by fixed monitoring stations located within the city of Paris. In total, Paris City counts 12 fixed monitoring stations measuring NO_2_, including 6 background stations and 6 traffic stations. To achieve this NO_2_ daily variability reconstitution, we followed a series of three steps. Firstly, each census block was assigned by AirParif to the monitoring station (named the 'index' monitor) best representing overall NO_2_ air quality within the census block, using clustering methods. Secondly, the ratio between daily NO_2_ concentrations for the 'index' monitor and its annual mean was computed for each 'index' monitor, so as to derive temporal series centred on 1. Thirdly, the annual concentration of NO_2_ modelled for each census block was multiplied by the daily variability ratio (obtained at the second step) for the associated 'index' monitor to compute daily concentration of NO_2_ for each census block. In this step, we hypothesized that daily variations of NO_2_ concentrations in a given census block would be similar to the daily variation of its 'index' monitor. Over the whole period of study, missing daily NO_2_ concentrations amounted to between 5% and 12%. Linear regression was used to substitute missing daily concentrations for each monitoring station using predicted values for the same day, based on mean daily concentrations for the available stations (separately for background and traffic stations).

**Table 1:** Descriptive statistics of NO_2_ concentrations (short and long term) across the study period (2004–2009).

Short term concentrations	Mean [CV%^†^]	Long term concentrations	Mean [CV%^‡^]
All blocks	52.59 [26.47%]	All blocks	53.21 [11.43%]
Least deprived blocks	52.78[25.29%]	Least exposed blocks	47.48 [4.76%]
Intermediate blocks	52.33 [26.66%]	Intermediate blocks	53.15 [2.92%]
Most deprived blocks	53.01 [26.99%]	Most exposed blocks	60.61 [7.18%]

^†^: expressed in μg/m^3^

^‡^CV% = coefficient of variation in %

### Socioeconomic index

To characterize socioeconomic status, we used an index developed at the census block scale for Paris, which is described elsewhere [26]. Briefly, a Principal Component Analysis (PCA) was used to select variables among 41 socioeconomic and demographic variables provided by the 2006 national census at census block level. According to the results of this Principal Component Analysis, 15 variables were most correlated with the first component, and thus selected to carry out a final Principal Component Analysis, where the reduced first component was used to calculate the socioeconomic index. Finally, hierarchical ascendant clustering was performed to gather census blocks into homogenous socioeconomic categories. Hierarchical clustering is an unsupervised clustering method which creates a hierarchy of classes (i.e. clusters), frequently used following a PCA or other data mining techniques. The purpose is to find a partition in *N* classes that either maximizes between-classes inertia or minimizes within-classes inertia. This inertia-based clustering criterion allows the creation of classes that are homogeneous in their composition and heterogeneous between one another. In our work, hierarchical clustering revealed 3 homogeneous socioeconomic categories numbered 1 (most privileged) to 3 (most deprived). The characterization of census blocks for these 3 categories is presented in S1 Table. About 13% (n = 126) of census blocks remained unassigned to a socioeconomic category because of being poorly inhabited (parks, recreation facilities, business and commercial districts, etc.). We did not, therefore, take these unclassified census blocks into account in the model (variables distributions were not modified: results not shown). In total, 866 IRIS (79,107 deaths) were analysed for the present study.

### Proven or likely confounders

Following the literature [20,27–31] dealing with the health effects of air pollution, several confounders were considered in our study.

- Daily temperature (maximum, mean and minimum), daily relative humidity (maximum, mean and minimum) and daily mean atmospheric pressure; the data were obtained from the Météo-France Montsouris station in Paris.
- Incidence rate of the weekly influenza case counts. Information on weekly influenza case counts in the Ile-de-France region (which includes the city of Paris as well as the whole surrounding metropolitan area) was obtained from the Inserm *Sentinelles* network (http://websenti.u707.jussieu.fr/sentiweb/).
- Holidays.

### Statistical analyses

Associations between daily NO2 concentrations and all-cause mortality were investigated using a case-crossover design [32]. Control days were selected using a monthly time-stratified approach: the study period was divided into monthly strata and control days were chosen from the same day of the week as the case day within each stratum [33]. For example, for a death occurring on a given weekday (e.g., a Monday), control days were the same days of the week throughout the rest of the month (thus, three or four days; here, all other Mondays of the same month). This control selection strategy takes account of time trends, seasonality, day of the week, temporal autocorrelation in exposure time-series, while avoiding overlap biases [33].

The statistical strategy was structured in 3 successive steps.

Firstly, associations between death events and ambient air pollution concentrations modeled by census block were estimated, adjusting for holidays, meteorological variables (the cubic B-spline function of the maximum daily temperature, the inverse of the humidity mean of the previous five days' deaths), and influenza epidemics, following the approach exposed below. Regarding temperature, we used a Cubic B-spline function of the maximum daily value which is recognised by the literature to have a strong short-term effect on mortality counts in comparison with the simple function of temperature [34–36]. Regarding humidity, we used the inverse of the mean of the previous five days' deaths (lag period 5 noted lag 0–5) [37] rather than the simple arithmetic mean, because it is a better fit. Finally, atmospheric pressure was not retained in the model because it was not significant, and AIC was not improved (see details in S2, S3 and S4 Tables).

In the second step, we tested the influence of the various lag periods between NO_2_ treated as a continuous variable, and short term mortality, by computing the mean of NO_2_ concentrations across the four to seven days prior to deaths (lag0-4 to lag0-7). Whatever the lag period of exposure we considered in the adjusted model, the short term effect of NO_2_ concentrations on mortality was statistically significant. However, we chose the 0–5 lag period which both gave a low AIC criteria and a low correlation with long-term NO_2_ exposure estimates.

Finally, in the third step, we assessed effect modification by age, sex, census block level socioeconomic status and long-term NO_2_ concentrations by testing interactions in the models.

Effect estimates of air pollution on the 0–5 lag period were expressed as the percent increase in risk of mortality (excess risk) associated with a 10 μg/m^3^ short-term increase in NO_2_, with corresponding 95% confidence intervals. All statistical analyses were performed using SAS version 9.3 (SAS Institute, Cary, NC).

## Results

The study included a total of 79,107 deaths among subjects above 35 years of age from 2004 to 2009. A majority (65%) occurred after the age of 75 and 38% beyond age 85; 53% of cases were female deaths.

Table 1 gives descriptive statistics of air quality, for both short-term NO_2_ concentrations (average of 5 days preceding death events) and long-term (average values over the study-period). Average NO_2_ concentration was 52.6 μg/m^3^ on the 5 days preceding death events (short-term exposure) and 53.2 μg/m^3^ over the study period (long-term exposure).

**Fig 1:**
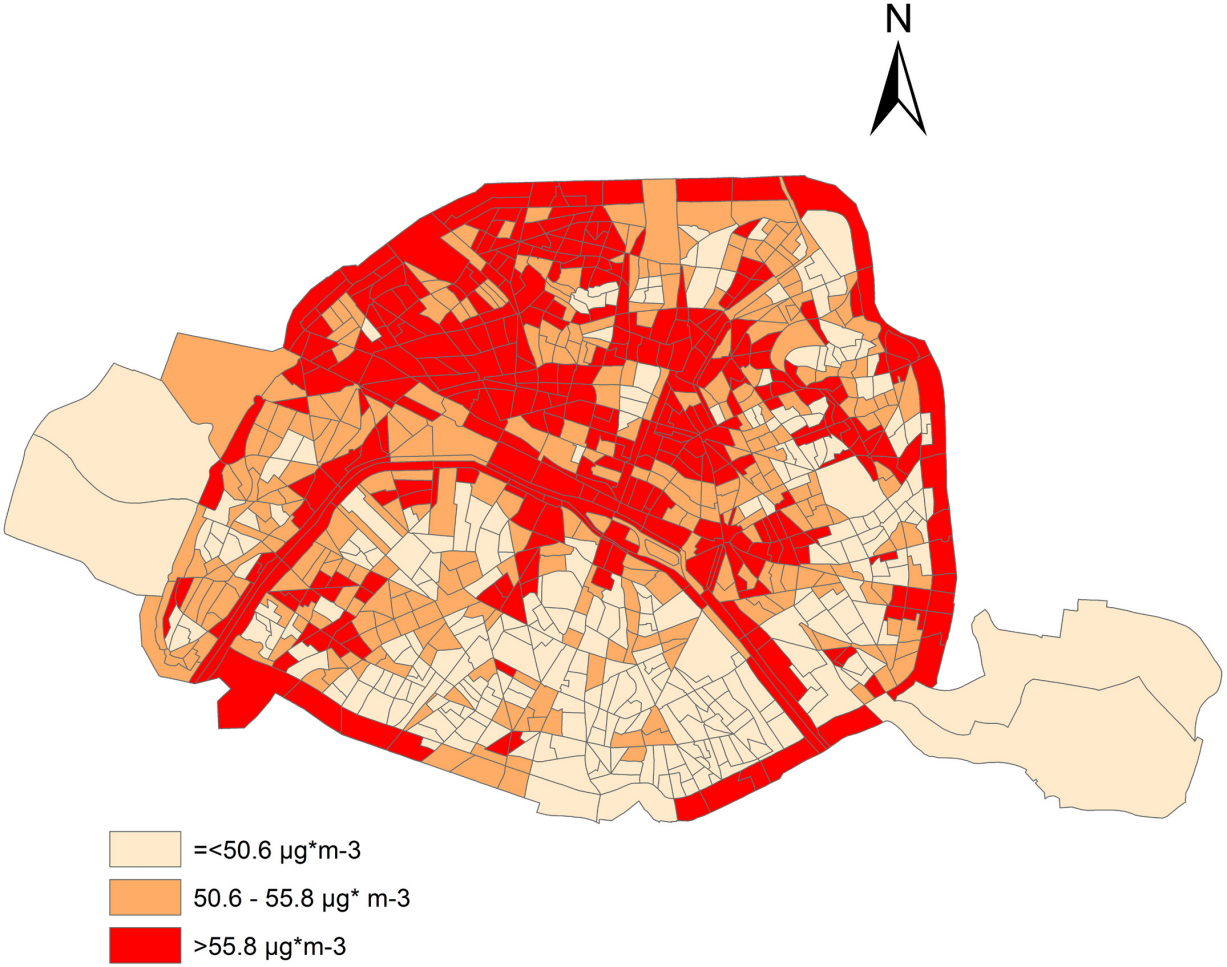
Socioeconomic categories in census block areas in Paris.

Socioeconomic categories: About 20% of subjects resided in socioeconomic category 1 census blocks (the most privileged), 55% in category 2, and 25% in category 3 (the most deprived). Fig 1 shows the residential distribution of these three socioeconomic categories in the city of Paris. The most deprived census blocks (category 3) are mainly located in the eastern and northern fringe of the city, i.e. along the *périphérique* (ring road), while census blocks characterized by a higher SES (categories 1 and 2) are found in the centre and western part of Paris. There was no evidence of differences in the short-term NO_2_ concentrations across the census blocks in three socioeconomic categories (Table 1).

**Fig 2:**
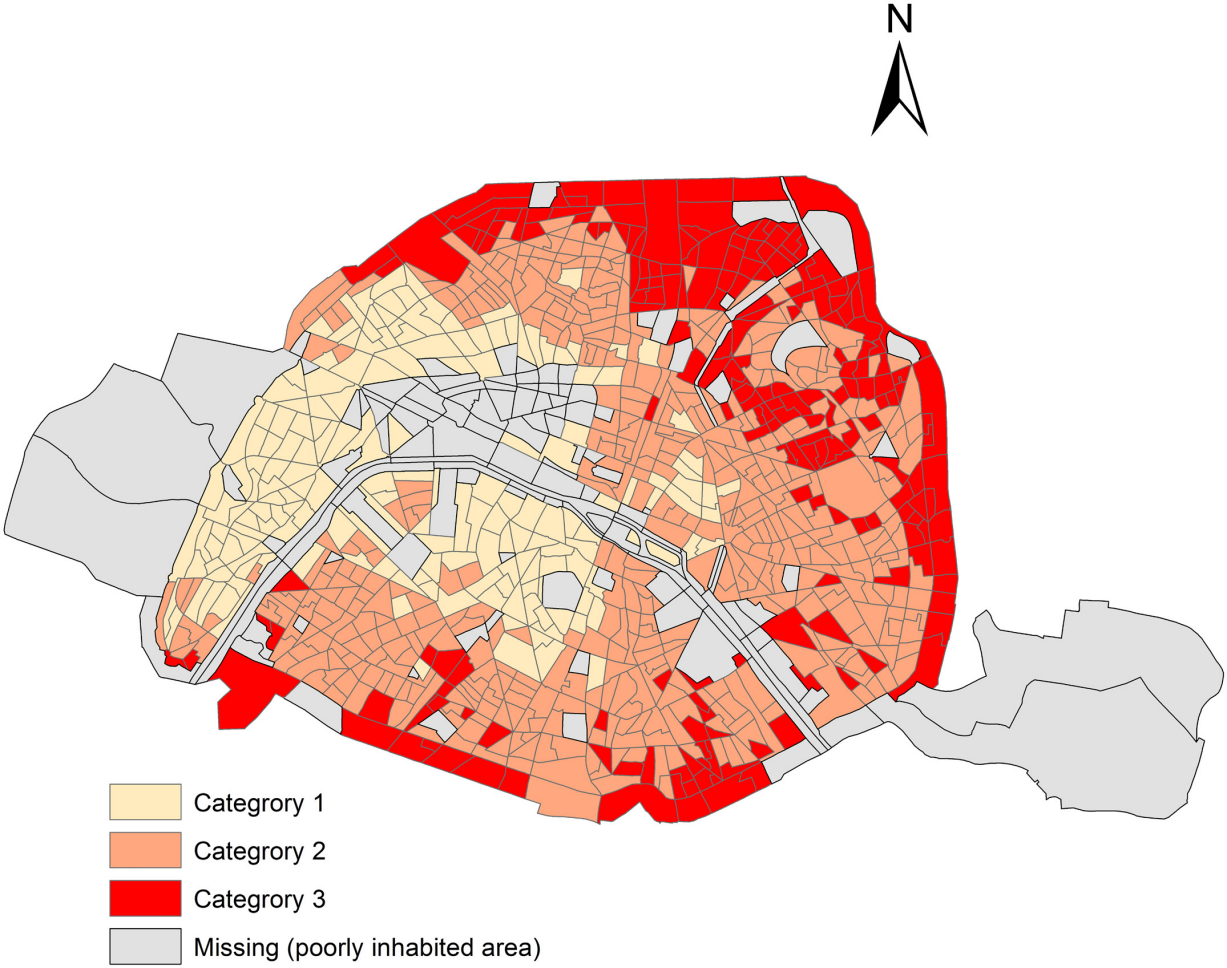
NO_2_ concentrations from 2002 to 2009, in census block areas within Paris.

NO_2_ long-term concentration: Fig 2 represents spatial distribution, at census block level, of long-term NO_2_ concentrations in the city of Paris. The most polluted areas are observed along the *périphérique* and the Seine river (next to major thoroughfares with high traffic volume) and in the north-western part of Paris.

All-cause mortality associated with short-term variations in NO_2_: The overall Excess Risk (ER) of all-cause mortality is equal to 0.94% (95%CI = [0.08;1.80]; p = 0.03) for a 10 μg/m^3^ increase in short-term NO_2_ concentrations (Table 2). Stratified analyses by individual characteristics revealed subgroups exhibiting a higher risk in relation with short-term NO_2_ concentrations (Table 2): elderly people (>85 years) and males. We also demonstrate that neighbourhood characteristics modify the effect of short term exposure to NO_2_. Subjects living in the lower socioeconomic category census blocks experience a higher NO_2_-related mortality (ER = 3.14%; 95%CI = [1.41, 4.90]) than in the most privileged ones (the interaction p-value is borderline significant = 0.07). When the privileged and middle socioeconomic categories were grouped, based on the similarity of their excess risk values, interaction between the short-term exposure to NO_2_ and SES became statistically significant (interaction p-value = 0.028).

**Table 2:** Excess risk of all-cause mortality associated with a 10-μg/m^3^ short-term NO_2_ increase, Paris, France, 2004–2009.

Variables	*n* (%)	Excess risk(%)^†^	95% CI	p value^‡^
**Total**	79,107 (100)	0.94	0.08, 1.80	**0.032**
**Age (year)**				
35–84	49,353 (62)	0.36	-0.72, 1.44	0.51
≥ 85	29,754 (38)	1.86	0.50, 3.24	**0.01**
**Sex**				
Female	41,774 (53)	0.22	-0.94, 1.38	0.71
Male	37,333 (47)	1.75	0.51, 3.00	**0.01**
**Census block socioeconomic categories**				
Category 1 (most privileged)	16,101 (20)	0.81	-1.01, 2.66	0.38
Category 2	43,582 (55)	0.04	-1.09, 1.18	0.95
Category 3 (most deprived)	19,424 (25)	3.14	1.41, 4.90	**0.00**
**Level of long-term NO**_**2**_ **exposure**				
1^st^ tertile: ≤ 50.6 μg/m^3^	29,894 (38)	0.06	-1.34, 1.47	0.94
2^nd^ tertile: 50.6–55.8 μg/m^3^	25,864 (33)	1.07	-0.30, 2.45	0.13
3^rd^ tertile: > 55.8 μg/m^3^	23,349 (30)	1.92	0.28, 3.59	**0.02**

^†^: Adjusted for maximum temperature (spline function), mean from lag 0 to 5 relative humidity (inverse function), incidence rate of influenza case counts, and holidays

^‡^: significant p-value in bold (p<5%)

**Fig 3:**
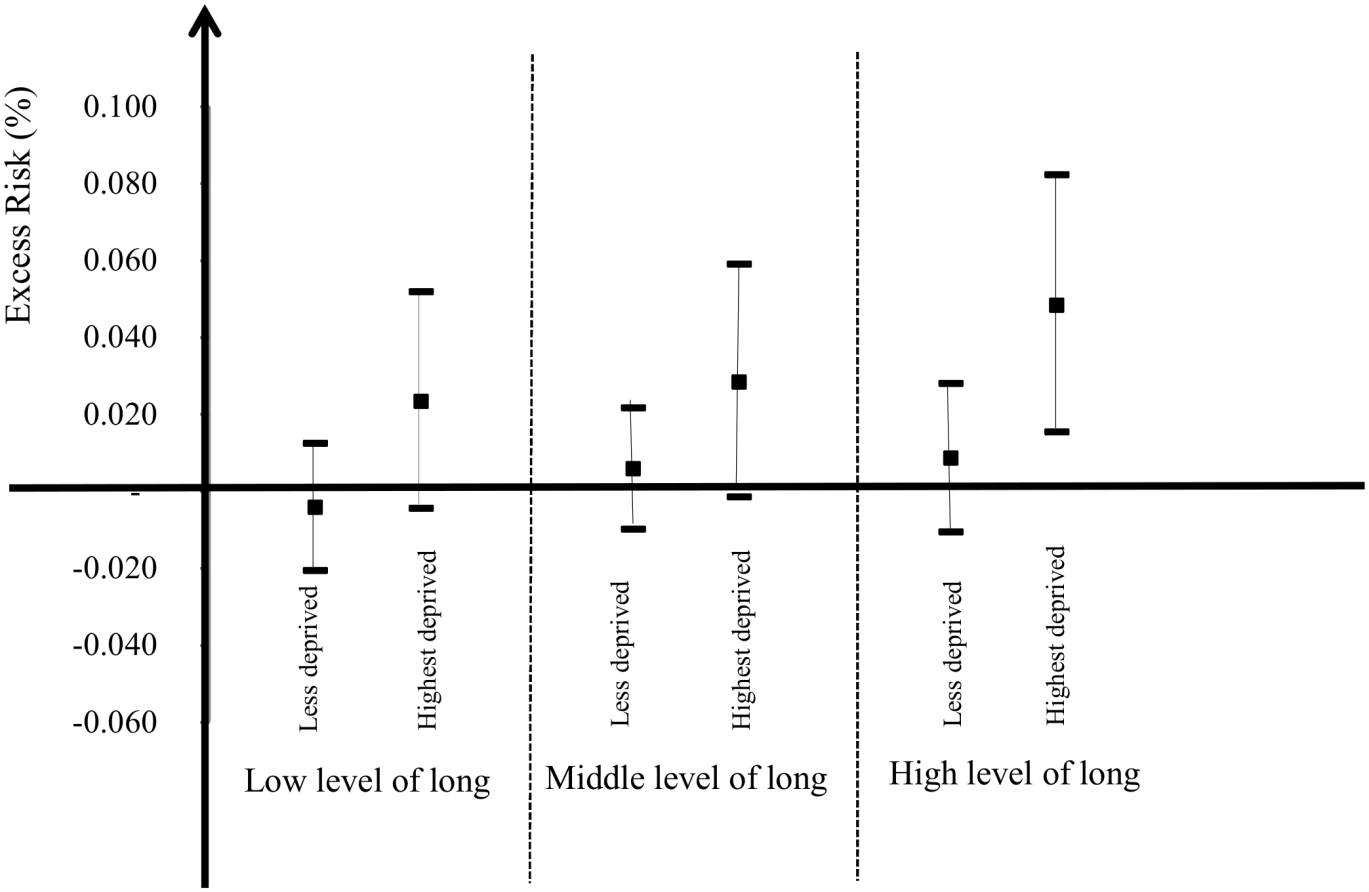
Excess risk of mortality associated with a 10-μg/m^3^ short-term NO_2_ increase and 95% confidence Interval, stratified by SES and long-term NO_2_ concentrations- Paris, France, 2004–2009.

People living in census blocks exhibiting the highest long-term exposure to NO_2_ had a higher risk of mortality in relation with short-term NO_2_ concentrations (ER = 1.92%; 95%CI = [0.28;3.59]) than the less-exposed census block (interaction p-value is borderline significant = 0.09). Further, Fig 3 shows the results of the combined effects of SES and long-term exposure to NO_2_, and reveals that the effect modification due to SES (the way SES affects the effect of short-term exposure on mortality) is significantly influenced by long-term NO_2_ values (the Chi-Square homogeneity-test which compared the 6 excess risks was borderline significant: p-value = 0.06). When the census blocks in the 1^st^ and 2^nd^ tertiles of long-term NO_2_ concentrations were grouped (because they had similar excess risks), the combined effect of SES and long-term NO_2_ concentrations became clearly significant (interaction p = 0.02), suggesting a higher risk of all-cause mortality related to changes in short-term NO_2_ concentrations in those census blocks that are both most deprived and have the highest long-term NO_2_ values (ER = 4.84%; 95%CI = [1.56; 8.24]).

## Discussion

We investigated the association between short-term variations of NO_2_ concentrations and all-cause mortality in the city of Paris between 2004 and 2009, with the aim of assessing whether this association was modified by population socioeconomic characteristics and/or long-term concentrations measured at census block level. We found an increase in all-cause mortality with a short-term 10-μg/m^3^ increase in NO_2_ during the 0–5 day lag period. Stronger associations were observed for subjects living in areas having low socioeconomic status. Our results also show an effect modification according to the combination of SES and long-term exposure to NO_2_: among the most deprived census blocks, excess risk is 4.84% (95%CI = [1.56;8.24]) when long-term average NO_2_ values are above 55.8 μg/m^3^ (the top tercile of the distribution) versus 2.56% (95%CI = [0.53;4.64]) when long-term average NO_2_ values are lower; corresponding figures in less deprived census blocks are 0.86% (95%CI = [-1.05;2.81]) and 0.12% (95%CI = [-1.02;1.26]).

Regarding the effect of socioeconomic status, our results are consistent with previous findings. Higher risks of all-cause mortality related to short-term exposure to NO_2_ or particulate matter (PM) have already been described among adults with poor socioeconomic characteristics such as: less qualified occupational activities [13, 17, 19], poor housing characteristics [19], low educational level [13, 17], and low socioeconomic index [13, 18].

Two main hypotheses may explain the increased health effect of air pollution among people in lower socioeconomic categories. Firstly, poorer subjects may be more exposed to higher levels of air pollutants due to their residential and/or occupational proximity to emitting sources. While this was described in the majority of studies [38, 39], some did report opposite findings [18, 40]. Within the city of Paris, we did not observe differences in annual mean NO_2_ between census blocks by socioeconomic category (Fig 1). Were there any such difference, it would be more in the direction of higher average values in better-off census blocks than in more deprived areas. As we showed elsewhere, such disparities are highly dependent on the historical social and urban make-up of each city [14, 41]. In our study, we assessed exposure to air pollution according to place of residence, which does yield some exposure misclassification. In their daily lives, individuals also encounter several micro-environments (at home, while commuting, at the workplace, etc.) and these do contribute substantially to their total exposure. Thus, exposure differential between socioeconomic categories could still be a reasonable hypothesis to explain our findings; indeed, indoor exposure at home varies across socioeconomic categories, with elevated concentrations of multiple pollutants in lower SES households [42]. Within the P.A.R.I.S. (Pollution and Asthma Risk: an Infant Study) infant cohort [43], a survey of indoor NO_2_ concentrations over a period of 7 days was undertaken in 196 homes. Concentrations measured in the homes of parents from low socioeconomic categories were, on average, higher than in homes from high socioeconomic categories (33.1 [13.3] μg/m3 vs. 25.9 [8.4] μg/m3) (C Roda, unpublished data, 2012). Furthermore, total exposure depends not only on ambient or indoor air concentrations, but also on the amount of time spent in these environments. Because they are much more likely to spend weekends and holiday periods away from Paris (e.g. at a second home in the countryside, or travelling), the time-weighted exposure of wealthier families to Parisian ambient air is likely to be lower than for deprived families, who take fewer holidays, less often and for shorter periods [44] as was noted in Rome [18]

Finally, the workplace micro-environment may also differentiate socioeconomic groups, and blue-collar workers and other modest social categories are more likely to be exposed to specific occupational exposures [45].

A second hypothesis invoked to explain the increased health effect of air pollution among lower socioeconomic categories is that groups with lower SES may be more vulnerable to the health effects of certain exposures because they experience poorer health for reasons directly related to their disadvantaged socioeconomic and psychosocial conditions [12]. Such populations, because of their limited economic and educational resources, may accumulate risk factors for chronic diseases. By this process, these populations would present 'a predisposition' to the development of health conditions as a result of any additional environmental insult. Such conditions may also hamper the continuation of occupational activities, a situation that will eventually lead to reduced income and, in turn, possibly to deteriorated health—and greater vulnerability to air pollution.

To our knowledge, no study has looked at whether the effect of short-term changes in air pollution on mortality differs according to the combination of long-term exposure to NO_2_ and socioeconomic status. The piecewise-constant proportional hazards model used in the New England study was successful in capturing both acute and chronic effects of PM2.5 [46]; however, it did not explore whether short-term effect estimates were influenced by long-term air pollution. An indication of a higher risk of mortality in association with short-term air quality variations in NO_2_ was observed, in our study, among the specific group composed of people living in the census blocks that were both most deprived and having higher long-term NO_2_ concentrations. A number of authors have shown that changes in air pollution levels increases mortality through a short-term harvesting effect, characterized by the precipitated death of frail subjects [7, 8]. This manifests as an increase in mortality within a few days of a pollution episode, followed by a period of decline in mortality during which the pool of people at highest risk of dying is reconstituted. Long-term exposure to air pollution has been described as a factor in reconstituting the frail subjects’ pool [8, 11]. Our results support the hypothesis that the rate at which this pool of at risk subjects is reconstituted may be higher in lower socioeconomic groups, where strong risk factors for mortality are more prevalent (smoking, pre-existing diseases, poorer access to health care). Moreover, long-term exposure to higher pollution is a cause of frailty through the development of chronic conditions—in particular such cardiovascular conditions as haemostasis alterations, atherosclerosis or hypertension [9, 47]. Further, SES might be associated with cumulative exposure to air pollutants—not only in ambient air but also indoors, both at home and in the workplace, as well as while commuting.

One limitation of our study is that socioeconomic status was assessed ecologically, at census block (rather than individual) level. The French census block is the smallest administrative unit for which socioeconomic and demographic information from the national census is available. The division of neighbourhoods into census blocks by the national statistical institute aims to maximize their homogeneity in terms of population size, socioeconomic characteristics, land use and zoning, thus reducing the risk of ecological bias. According to Krieger et al. [48], the census block is an adequate geographical scale for the assessment of social inequalities.

Our results should not be interpreted in causal terms. Rather, nitrogen dioxide levels are indicators of proximity to emission sources associated with industrial combustion processes, urban heating and petrol or diesel powered traffic [31]. In the city of Paris, and in general in the Ile-de-France region (whose economy shifted, over the last three decades of the 20^th^ century, from industry to services) NO_x_ emissions are mainly associated with traffic—as clearly illustrated in Fig 1B, where higher annual NO_2_ levels match areas of dense traffic. We cannot rule out the possibility that the associations observed could be explained by other air pollutants having the same sources. Several studies have shown a strong correlation between traffic soot/ultrafine particles and NO_2_ [33, 38]. For those Paris city monitoring stations having PM_10_ and PM_2.5_ concentrations available for the 2002–2009 period, we computed the correlation between NO_2_ and PM, with no data on ultrafine particles. This correlation ranged from 0.45 to 0.74 for PM_10_ and between 0.51 and 0.66 PM_2.5,_ by monitoring site.

Our findings suggest a higher risk of short-term all-cause mortality among men than among women. A few studies have looked at gender as a potential effect-modifier for the association between NO_2_ or PM and mortality, reaching partially inconsistent conclusions [13, 20, 23, 49]. These results need further investigation, in particular to better understand whether the effect modification by gender could be explained by socially-derived gendered exposures and/or by biological differences (e.g. hormonal status).

## Conclusions

In conclusion, the present study provides evidence that socioeconomic status has an effect on the association between short-term exposure to ambient air NO_2_ and all-cause mortality in the city of Paris, and interacts with long-term exposures. Because there is not wide variation between NO_2_ concentrations across the census blocks in the three socioeconomic categories, differential vulnerability is likely to be the most probable explanation of our finding. Other studies should be conducted to further explore these complex processes.

## Supporting Information

S1 Table Description of socioeconomic categories. (DOCX)

S2 Table Model parameters for confounders. (DOCX)

S3 Table Knots for spline effect of maximum daily temperature. (DOCX)

S4 Table Basics details for spline effect of maximum daily temperature. (DOCX)
