# Correction: Neighbourhood Characteristics and Long-Term Air Pollution Levels Modify the Association between the Short-Term Nitrogen Dioxide Concentrations and All-Cause Mortality in Paris

**DOI:** 10.1371/journal.pone.0150875

**Published:** 2016-03-01

**Authors:** Séverine Deguen, Claire Petit, Angélique Delbarre, Wahida Kihal, Cindy Padilla, Tarik Benmarhnia, Annabelle Lapostolle, Pierre Chauvin, Denis Zmirou-Navier

The images for Figs 1 and 2 are incorrectly switched. The image that appears as Fig 1 should be Fig 2, and the image that appears as Fig 2 should be Fig 1. Please see corrected Figs 1 and 2 here. The figure captions appear in the correct order.

**Fig 1:**
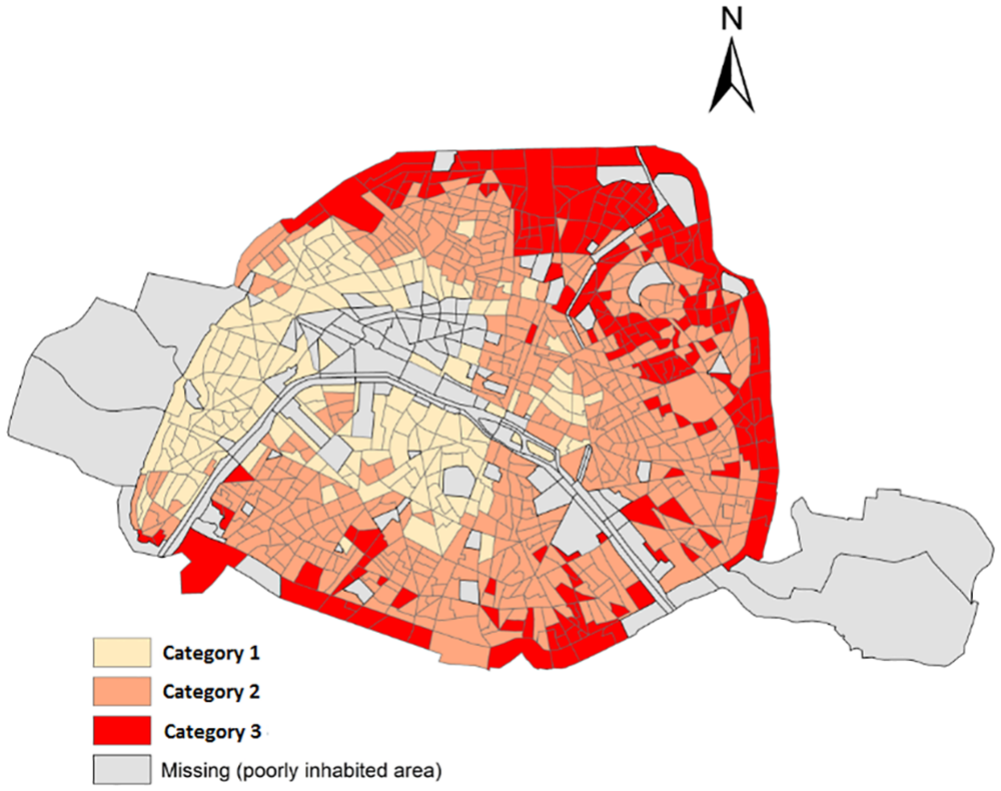
Socioeconomic categories in census block areas in Paris.

**Fig 2:**
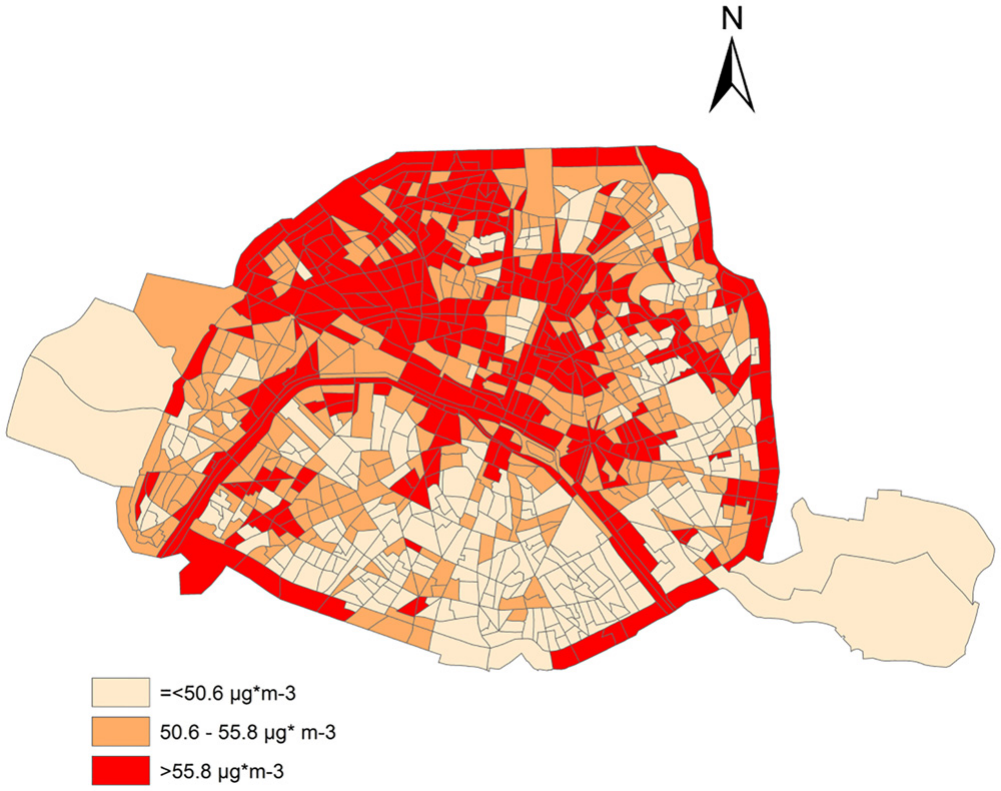
NO_2_ concentrations from 2002 to 2009, in census block areas within Paris.

There are errors in Fig 3. Please see the corrected Fig 3 here.

**Fig 3:**
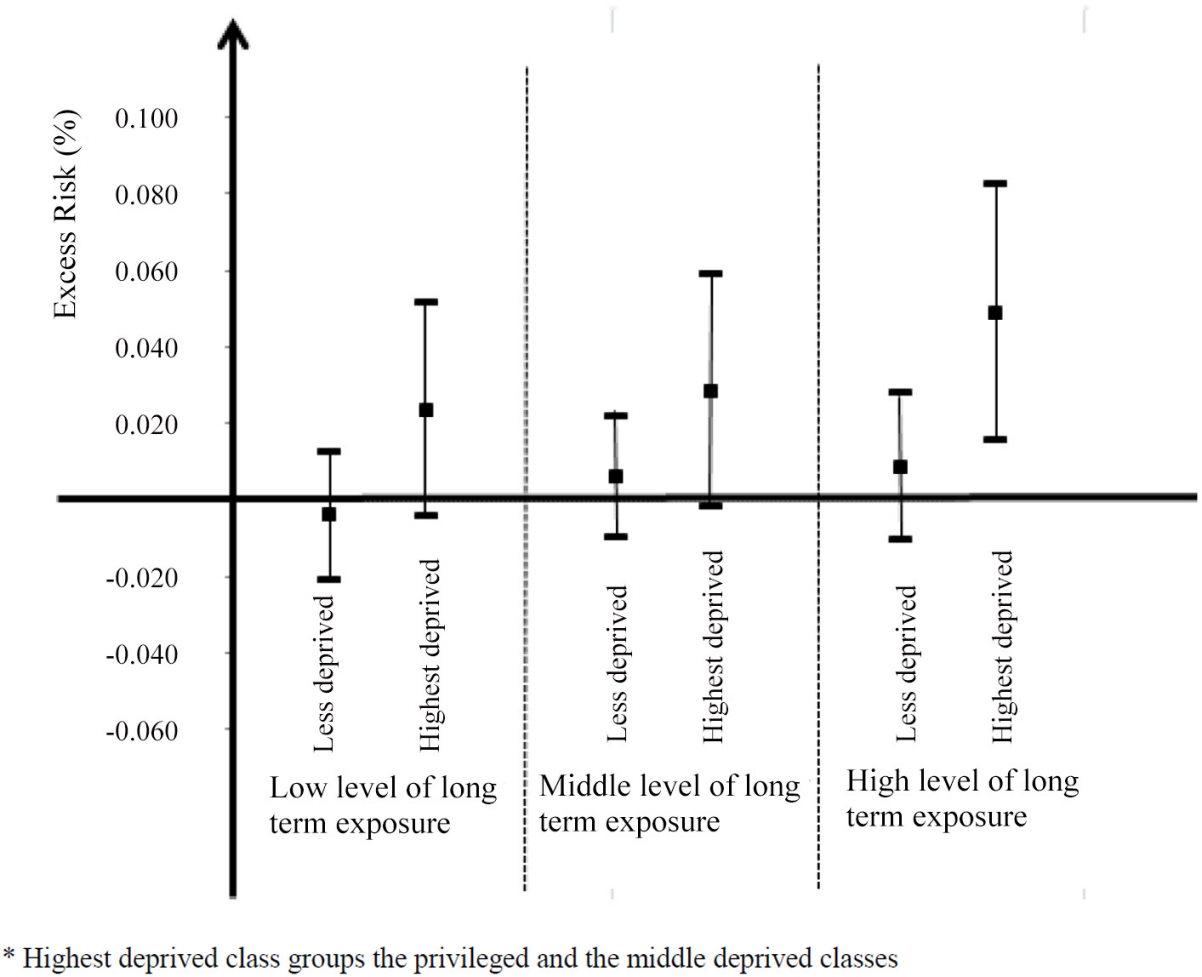
Excess risk of mortality associated with a 10-μg/m^3^ short-term NO_2_ increase and 95% confidence Interval, stratified by SES and long-term NO_2_ concentrations- Paris, France, 2004–2009.
