# Supplementary material for: Neighbourhood Characteristics and Long-Term Air Pollution Levels Modify the Association between the Short-Term Nitrogen Dioxide Concentrations and All-Cause Mortality in Paris
Source: PLoS One. 2015 Jul 21;10(7):e0131463. doi: 10.1371/journal.pone.0131463 (PMC4510557; doi:10.1371/journal.pone.0131463)
Supplement: S1 Table — (DOCX) [file pone.0131463.s001.docx]

**SUPPLEMENT S1**

**TITLE**

Neighbourhood characteristics and long-term air pollution levels modify the relation between the short-term variability of nitrogen dioxide concentrations and all-cause mortality in Paris.

**Table; Supplemental Material. Description of socioeconomic categories.**

| **Socioeconomic categories** | **Description** |
| --- | --- |
| Category 1 | Census blocks with a high median income and high percentages of housings ≥ 100 m², self-employed, craftsmen, managers, and people with a higher educational degree |
| Category 2 | Census blocks with high percentages of managers and people with a higher educational degree |
| Category 3 | Census blocks with high percentages of non-graduates, blue-collar workers, employees, subsidized housings, single-parent families, unemployed, people with basic or intermediate general or vocational qualifications, non-owners, and foreigners |
