# Supplementary material for: Neighbourhood Characteristics and Long-Term Air Pollution Levels Modify the Association between the Short-Term Nitrogen Dioxide Concentrations and All-Cause Mortality in Paris
Source: PLoS One. 2015 Jul 21;10(7):e0131463. doi: 10.1371/journal.pone.0131463 (PMC4510557; doi:10.1371/journal.pone.0131463)
Supplement: S2 Table — (DOCX) [file pone.0131463.s002.docx]

**Supplement S2**: Model parameters for confounders

| **Variable** | **Function** | **β-coefficient** | **Standard Error** | **p-value** |
| --- | --- | --- | --- | --- |
| Maximum of the daily temperature | Cubic B-spline^*^ | **β**_1_=2.74 | 1.27 | 0.03 |
|  |  | **β**_2_=1.15 | 1.01 | 0.19 |
|  |  | **β**_3_=1.23 | 0.99 | 0.15 |
|  |  | **β**_4_=1.23 | 1.00 | 0.16 |
|  |  | **β**_5_=1.22 | 0.98 | 0.15 |
|  |  | **β**_6_=1.5 | 1.06 | 0.11 |
| Mean of daily relative humidity | Inverse of the humidity mean of the previous five days' deaths | 7.15 | 3.41 | 0.04 |
| Influenza count | Incidence rate | 1.2.10^-4^ | 7.98.10^-5^ | 0.11 |
| Holidays | Dummy variable | 0.029 | 0.010 | 0.006 |

^*^ Details are given in supplement S3 and supplement S4
