# Supplementary material for: Neighbourhood Characteristics and Long-Term Air Pollution Levels Modify the Association between the Short-Term Nitrogen Dioxide Concentrations and All-Cause Mortality in Paris
Source: PLoS One. 2015 Jul 21;10(7):e0131463. doi: 10.1371/journal.pone.0131463 (PMC4510557; doi:10.1371/journal.pone.0131463)
Supplement: S3 Table — (DOCX) [file pone.0131463.s003.docx]

**Supplement S3**: Knots for spline effect of maximum daily temperature

| Knots’ number | Threshold of maximum daily temperature (expressed in degree Celsius - °C) |
| --- | --- |
| 1 | -24.55 |
| 2 | -14.22 |
| 3 | -3.90 |
| 4 | 6.42 |
| 5 | 16.75 |
| 6 | 27.07 |
| 7 | 37.40 |
| 8 | 47.72 |
| 9 | 58.05 |
