# Supplementary material for: Neighbourhood Characteristics and Long-Term Air Pollution Levels Modify the Association between the Short-Term Nitrogen Dioxide Concentrations and All-Cause Mortality in Paris
Source: PLoS One. 2015 Jul 21;10(7):e0131463. doi: 10.1371/journal.pone.0131463 (PMC4510557; doi:10.1371/journal.pone.0131463)
Supplement: S4 Table — (DOCX) [file pone.0131463.s004.docx]

**Supplement S4**: Basics details for spline effect of maximum daily temperature

| β-coefficients | [Support range]^*^ | | Corresponding knots |
| --- | --- | --- | --- |
| **β**_1_ | [-24.55 | ; 6.42] | 1-4 |
| **β**_2_ | [-24.55 | ; 16.75] | 1-5 |
| **β**_3_ | [-14.22 | ; 27.07] | 2-6 |
| **β**_4_ | [-3.9 | ; 37.4] | 3-7 |
| **β**_5_ | [6.42 | ; 47.72] | 4-8 |
| **β**_6_ | [16.75 | ; 58.05] | 5-9 |

^*^ expressed in degree Celsius - °C
